# Supplementary material for: Effects of Ramadan on clinical visits at a primary health center in a Rohingya refugee camp in Bangladesh
Source: PLOS Glob Public Health. 2025 Jun 18;5(6):e0004764. doi: 10.1371/journal.pgph.0004764 (PMC12176199; doi:10.1371/journal.pgph.0004764)
Supplement: Attachment — Submitted filename: Point-by-Point Response Letter.pdf [file pgph.0004764.s003.pdf]

13 August 2024
Manuscript #: PGPH-D-24-00196

TITLE: The effects of Ramadan on clinical consultation frequency in a Rohingya refugee camp, Bangladesh

Point-by-Point Response Letter

We appreciate the reviewer's positive assessment of our work and the constructive feedback provided. In response to the reviewer's suggestions, we have made extensive revisions to both the analysis and the discussion sections. While the core findings of the study remain consistent with our original submission, the revised manuscript now demonstrates enhanced clarity and logic in the presentation of results. We have addressed each of the reviewer's comments in detail, and the revisions are outlined as follows:

REVIEWER 1

1. This is essentially an extraction of quantitative data from a database that was not created for this research but for an administrative purpose. There is a complete lack of any qualitative information, either from the population served or the healthcare workers involved that could have informed the discussion, the latter which is based entirely on comparing the quantitative data, somewhat speculative, with those of other studies. Some of those other studies had at least a qualitative component

Our response: Our study is designed as a quantitative retrospective analysis with the specific aim of extracting and analysing quantitative data from an existing administrative database. This focus was chosen to provide a comprehensive statistical overview of the patterns and trends within the patient data we have collected. While we acknowledge the value that qualitative insights can bring to a comprehensive understanding of healthcare outcomes, the scope of our current study was explicitly defined to be quantitative. Incorporating qualitative data would require a fundamentally different study design, including data collection methods like interviews or surveys with patients and healthcare workers. These methodologies were beyond the scope and resources of our current project, which was constrained to analysing existing quantitative data. Future research could certainly benefit from including qualitative components to provide a more holistic understanding of healthcare dynamics in this context.

2. Is the study clinic the only one providing healthcare to the population 24h? Are there any other health service providers that could have biased the clinic attendance in this service? (E.g. in many camps there is a setup that out of hours care is provided by a different organisation).

Our response: As described in the introduction, our facility is the only primary health center in Camp 14 equipped with Basic Emergency Obstetric Care services. Prior to our operations starting in February 2020, the other five healthcare providers in Camp 14, including a few fixed health posts and occasional mobile clinics operated by other organizations, offer irregular services due to funding shortages and unstable financial support, which may have minimal impact on clinic attendance. At that time, Médecins Sans Frontières (MSF) did not provide such services, although they operated a Primary Health Care (PHC) facility in Camp 14 that offered Antenatal Care (ANC) and Postnatal Care (PNC) services but did not handle natural deliveries. We have included this information in the revised manuscript (Lines 36-38, 46-50).

3. (A) Some clearer description of the study design would be helpful, e.g. purpose, objectives, definition of study groups, definition of study type. It should be made clearer that this is a retrospective cohort study from a data collected for a more administrative purpose. (B) The reason for selecting the five health events is not clearly described – was this because these were the most common? Or thought of the most relevant for Ramadan related health conditions?

Our response: We fully reflected the reviewers' comments and elaborated methods as per reviewer's advice. (A: Line 71-168, B: 143-149,)

4. Whilst the number of datasets is large, the group sizes are highly discrepant. It would be helpful to provide a statistical power calculation for the expected/encountered outcomes.

Our response: As suggested by another reviewer, we have segmented the study period into three distinct phases: before Ramadan, during Ramadan, and after Ramadan in accordance with previous literature (Şimşek P, Yadigaroğlu M, Katipoğlu B, Şahin A, Topbaş M, Gündüz A. Effect of Ramadan on Emergency Department Attendances: A Retrospective Study. Eurasian J Emerg Med. 2021 Dec;20(4):219-223). In our analysis, the Ramadan period is defined as April 23 to May 23, 2020, excluding May 24-26 (Eid-ul-Fitr Public Holiday) to prevent dilution of Ramadan effects. The period before Ramadan was designated from March 23 to April 22, 2020, while the period after Ramadan extended from May 27 to June 26, 2020. Each of these periods covers 31 days, addressing the previous concern regarding unequal group sizes. We hope this adjustment resolves the issue and provides a more balanced comparison.

5. Why is there such a predominance of female attendees at the clinic? Does this reflect the camps demographics? Did you include children in the data, and what do you know about their adherence to Ramadan? Children carry a large proportion of gastrointestinal conditions. Did you analyse this to avoid potential bias?

Our response: The predominance of female attendees at the clinic reflects the camp's demographics to some extent. According to the population factsheet provided by the Government of Bangladesh and UNHCR, as of March 2023, the total Rohingya population is 960,539 individuals, of which 52% are children and 51% are female. During the daytime, females do not work and often seek medical services while bringing their children to the facility. This may be due to the fact that many men work during the day, while women tend to stay at home, leading to a higher tendency for mothers to seek medical care along with their children.

Moreover, our facility is staffed with female obstetricians and midwives, providing comprehensive perinatal care, which likely contributes to the higher number of female patients. Women may feel more comfortable seeking medical care from female healthcare providers, particularly for gynecological and obstetric services. This specialized care attracts more women to our clinic. It is indeed true that female visits were higher among the Rohingya during the entire study period. However, the primary objective of our study was to determine whether females or males were more likely to visit the clinic during Ramadan compared to before or after Ramadan. Given this focus, we decided not to elaborate on the overall number of female visits, as it would divert from the main scope of our analysis. We observed that the proportion of female patients remained stable during Ramadan compared to the period before and after Ramadan. This observation is discussed in detail in the revised manuscript (Line 326-332). Additionally, following the reviewer's suggestion, we have excluded children younger than 15 years old from the analysis, as fasting is not mandatory for them (Line 133-135).

6. Having qualitative data from the same study population would be helpful to enlighten and give meaning to the data, rather than estimating the context from other studies. I appreciate this can be difficult in a camp situation. Alternatively, you could conduct focus groups with the health workers involved to inform your findings.

We appreciate your suggestion to include qualitative data to provide deeper insights and context to our findings. As previously mentioned, we understand that qualitative data can significantly enhance the interpretation and meaning of quantitative results, especially in complex settings like ours.

Unfortunately, due to the current constraints of our study, particularly the logistical challenges and limited access to the study population within the camp, we are unable to conduct new interviews or focus groups at this time. However, we have attempted to enrich the context of our findings by consulting with healthcare professionals and experts familiar with the camp's healthcare environment. Their insights and expertise are reflected in the discussion section of our revised manuscript. This approach, while not a direct substitute for qualitative data, has provided valuable context and helped to inform our analysis.

7. The last paragraph of the discussion is a bit out of context, as you present no data around reproductive health. You may consider removing this or reframing this.

Our response: Thank you for your feedback. We have reframed the last paragraph to align better with the study's scope and emphasize the importance of addressing reproductive health needs. We highlight that many pregnant women in the camps do not receive antenatal and postnatal check-ups at the frequency recommended by WHO, and there is a shortage of 24/7 health centers providing emergency obstetric and newborn care. Therefore, promoting facility-based deliveries, as well as antenatal and postnatal check-ups, remains crucial for improving maternal and child health outcomes. We have included this information in the revised manuscript (Lines 343-349).We believe this revised paragraph now better fits the context of our study and addresses the reviewer's concern effectively.

8. The limitations should include a discussion about the limited interpretability of quantitative data.

Our response: Thank you for your suggestion. We have added a statement in the limitations section acknowledging that qualitative data can significantly enhance the interpretation and meaning of quantitative results. However, we were unable to extend our quantitative research to include qualitative methods due to logistical constraints and limited resources. We have reflected reviewers' comments (Line 364-367).

9. Figure 1 deserves more explanation – there are huge fluctuations in the number of health events throughout the year, whilst the change during Ramadan appears very small. Why is that? What events do the peaks and troughs represent?

Our response: The fluctuations in the number of health events throughout the year reflect a complex interplay of various factors. In 2020, the onset of the COVID-19 pandemic led to significant societal changes. Additionally, internal reports indicate that events such as the installation of barbed wire around the refugee camp, periods of heightened security concerns due to fires, heavy raining and other incidents contributed to these fluctuations, particularly during the autumn of that year. Further contributing factors include the opening of a new clinic nearby and changes in the healthcare services available locally, which impacted clinic attendance patterns. These elements collectively explain the observed variability in the number of clinic visits throughout the year. Identifying specific reasons for each peak and trough remains challenging due to the multitude of contributing factors. We have included a more detailed explanation in the revised manuscript to provide clarity on these points (Lines 273-281).

REVIEWR 2

1. While the current study focuses on the effects of Ramadan on clinical consultation frequency in the Rohingya refugee camp, it does not reference any additional literature that explores similar effects in diverse healthcare settings, and populations.

Our response: We appreciate the reviewer's suggestion to include references to additional literature that examines the effects of Ramadan on healthcare utilization in different settings. To address this, we have incorporated findings from a relevant study that provides a broader context to our results: Khojah et al. (2023) conducted a three-year retrospective study in a Muslim-majority country, analyzing the demographics and clinical presentations of patients visiting the emergency department during Ramadan. They observed a decrease in overall ED visits and trauma cases, while gastrointestinal issues increased during Ramadan. This study supports our findings of reduced health events and increased gastrointestinal problems during Ramadan in the Rohingya refugee camp. We have included this reference in the revised manuscript (Line 261-263) to provide a more comprehensive overview of the impact of Ramadan on healthcare utilization across different settings.

2. Also, the authors state in the limitations section that the analysis includes patients from the host community, whose religion is not surveyed and may not be as consistent as the Rohingya population. This could introduce variability in the data and affect the generalizability of the findings. Ramadan is associated with a specific religious practice and denomination and the majority of Rohingya refugee population is Muslim so what is the point of including non-muslim population in the study?

Our response: In line with the reviewer's suggestion, we have excluded data from the host community to ensure the study focuses exclusively on the Muslim-majority Rohingya population, thereby reducing variability related to religious practices. Additionally, we excluded children under the age of 15, who are not required to fast during Ramadan, from our analysis. These adjustments aim to improve the consistency of the study population and enhance the accuracy and generalizability of our findings concerning the impact of Ramadan on health event rates. These changes have been reflected in the revised manuscript (Lines 132-135).

3. The inclusion of host population in the study is not sufficiently explained and documented. Limiting the sample to the Rohingya refugee population only would be more accurate, in line with the scope and objective of the study.

Our response: As mentioned in our previous response, we have excluded data from the host community to ensure the study focuses solely on the Rohingya refugee population.

4. The authors state that the control period is from January 2020 to December 2020, excluding certain dates during Ramadan and other public holidays. However, they do not explicitly state whether the control period is before or after Ramadan. I suggest to specify this point, which may have an effect on the frequency of consultation and the types of health events reported. Time series analysis might offer a more nuanced understanding of the consultation patterns.

Our response: Thank you again for this valuable comment. As per your suggestion, we have segmented the study period into three distinct phases: before Ramadan, during Ramadan, and after Ramadan in accordance with previous literature (Şimşek P, Yadigaroğlu M, Katipoğlu B, Şahin A, Topbaş M, Gündüz A. Effect of Ramadan on Emergency Department Attendances: A Retrospective Study. Eurasian J Emerg Med. 2021 Dec;20(4):219-223). In our analysis, the Ramadan period is defined as April 23 to May 23, 2020, excluding May 24-26 (Eid-ul-Fitr Public Holiday) to prevent dilution of Ramadan effects. The period before Ramadan was designated from March 23 to April 22, 2020, while the period after Ramadan extended from May 27 to June 26, 2020. Each of these periods covers 31 days. Given that we have restricted the analysis period to three months by segmenting it into before, during, and after Ramadan, time series analysis is not appropriate for this study (Line 122-127, Figure 1).
