# Supplementary material for: Effects of Ramadan on clinical visits at a primary health center in a Rohingya refugee camp in Bangladesh
Source: PLOS Glob Public Health. 2025 Jun 18;5(6):e0004764. doi: 10.1371/journal.pgph.0004764 (PMC12176199; doi:10.1371/journal.pgph.0004764)
Supplement: Attachment — Submitted filename: 250122_Point-by-point response to Reviewers comment2.docx [file pgph.0004764.s004.docx]

*27 December 2024*

*Manuscript #: PGPH-D-24-00196*

*TITLE: The effects of Ramadan on clinical consultation frequency in a Rohingya*

*refugee camp, Bangladesh*

**Point-by-Point Response Letter**

Thank you for your continued feedback and constructive comments on our manuscript. In this second response, we have carefully considered the reviewer’s suggestions and made further refinements to the analysis, methodology, and discussion sections. These revisions aim to improve the clarity and robustness of our manuscript while addressing the specific points raised. Below, we provide a detailed response to each of the reviewer’s comments.

REVIEWER 1

1. I think you need to highlight very prominently, in the study design section as well as limitations, that your data were NOT collected for research or publication purposes, BUT for administrative reasons.

Our response:	We appreciate this important observation. To address this, we have clarified in the study design section that the data were primarily collected for administrative and operational purposes. Specifically, we have added the following sentence at Line 120-123 in the revised manuscript: *"To assist with clinical monitoring and reporting to administrative authorities, a database was introduced at the PHC in March 2019. The data collected through this system were primarily intended for monitoring clinic activities and reporting to stakeholders, rather than for research or publication."* Additionally, we added at Line 429-432 in the Limitation section: *"the data were collected primarily for clinical monitoring and administrative reporting purposes, rather than for research, which may limit the depth of analysis. "*

2. I still struggle with your interpretation of the data which remains very speculative, in particular the section from line 468 where you provide no evidence that such reasons applied. I notice there were higher attendances for new appointments/routine health visits in the pre-Ramadan period as compared to the post Ramadan phase - could it be possible that patients were planning ahead bringing such appointments forward anticipating the holidays? Could you break these data down into weeks/months rather than 3 periods? Your numbers should allow for that. Please undertake a more systematic literature search to underpin your interpretations of your data, if possible from the same setting and time period, including 'grey literature' such as reports by organisations.

Our response: Thank you for your valuable feedback. While the editor referred to Line 468, which appears to be the reference section in the manuscript, we supposed that it likely corresponded to the observation that new visits were higher during the pre-Ramadan period.

We agree that the observed increase in visits during the pre-Ramadan period may reflect, at least in part, preparatory behavior among patients. These behaviors could include confirming health status, completing necessary treatments, and optimizing medications to mitigate potential health risks during fasting.

To further explore your suggestion, we conducted sensitivity analyses using three different options for defining the pre- and post-Ramadan periods:

1. **Option 1**: 14 days before Ramadan (BR) – Ramadan (R) – 14 days after Ramadan (AR);

2. **Option 2**: The original approach (31 days for each period);

3. **Option 3**: 2 months before Ramadan (BR) – Ramadan (R) – 2 months after Ramadan (AR).

Across these approaches, the analysis showed that the patterns remained consistent, with new visits being consistently higher during the BR period across all options. However, we observed some variability in ARI trends. For instance, in Option 3, ARI visits were higher in the 2 months before Ramadan compared to Ramadan, likely reflecting seasonal effects as the pre-Ramadan period in this option corresponds to cooler weather, which may exacerbate respiratory infections. This suggests that extending the time periods may lead to results that are more reflective of seasonal trends than Ramadan-specific effects.

For NCDs, which represent a category of non-urgent conditions, we observed that the proportion of visits increased progressively as the pre-Ramadan period was extended from 2 weeks to 1 month and then to 2 months. This trend may reflect the preparatory behavior of patients seeking to manage chronic conditions or obtain routine care in anticipation of fasting. However, it remains challenging to determine precisely at what point this preparatory behavior begins to influence visit patterns. Further research, such as patient interviews or qualitative studies, would be required to explore this phenomenon and confirm its relevance to visit patterns.

However, it is important to note that there are no universally accepted cut-off points for defining the pre- and post-Ramadan periods in such studies. These periods can range from one week to several months, making any choice somewhat arbitrary. Selecting a shorter period, such as seven or fourteen days, could capture immediate changes but risks missing variability associated with longer-term effects. On the other hand, longer periods, such as two to three months, risk diluting Ramadan-specific effects with broader seasonal or long-term trends. In terms of statistical concept, shorter windows reduce the total number of data points in the before/after segments, which have limited statistical power. Conversely, very long windows may dilute the specific Ramadan effect by blending in too much baseline variability beyond seasonality (*Hulley, S. B., Cummings, S. R., Browner, W. S., Grady, D. G., & Newman, T. B. (2013). Designing Clinical Research (4th ed.). Lippincott Williams & Wilkins; Shadish, W. R., Cook, T. D., & Campbell, D. T. (2002). Experimental and Quasi-Experimental Designs for Generalized Causal Inference.; Vandenbroucke, J. P., Von Elm, E., Altman, D. G., et al. (2007). Strengthening the Reporting of Observational Studies in Epidemiology (STROBE): Explanation and Elaboration. Epidemiology, 18(6), 805–835.)*

We believe that the most suitable analytical approach to compare three periods without relying on specific cut-off points would be interrupted time series analysis. However, our dataset contains too few event-specific data points to apply this method effectively (Penfold RB, Zhang F. Use of interrupted time series analysis in evaluating health care quality improvements. *Acad Pediatr*. 2013;13(6 Suppl):S38-S44 ; Wagner AK, Soumerai SB, Zhang F, Ross-Degnan D. Segmented regression analysis of interrupted time series studies in medication use research. *J Clin Pharm Ther*. 2002;27(4):299-309).

Our original approach aligns with existing literature, where studies such as those by Şimşek et al. (2021) and similar investigations have often used one-month periods before and after Ramadan for comparison. In addition, because Ramadan itself spans approximately 29–30 days, using a one-month window before and after Ramadan provides symmetrical and consistent measurement periods, thereby simplifying the interpretation of any observed changes.

Considering the factors discussed above, we believe our original choice of a one-month period aligns well with both clinical and practical considerations, as it reflects the duration of Ramadan and captures potential behaviors or trends associated with preparation and recovery.

We have incorporated this perspective into the Method section (Line 161-168) and the Discussion section (Line 308-323) of the revised manuscript, clarifying the rationale behind our one-month framework and addressing the potential limitations of alternative approaches.

3. Some minor points: Should it be 'sex' instead of 'gender'? I suggest to not make reference to 'Burma' because of the colonial connotations.

Our response:

1) Regarding the term "gender": we have carefully reviewed the manuscript and replaced instances of "gender" with "sex" where appropriate, as the data collected pertains to biological differences rather than social constructs.
2) We acknowledge the colonial connotations associated with the term "Burma." Accordingly, we have removed the reference to "Burma" and updated the text to refer only to "Myanmar." This ensures alignment with respectful and current usage. Specifically, the phrase "previously called Burma" has been omitted. We appreciate your thoughtful feedback on these matters.
