# Supplementary material for: Effects of Ramadan on clinical visits at a primary health center in a Rohingya refugee camp in Bangladesh
Source: PLOS Glob Public Health. 2025 Jun 18;5(6):e0004764. doi: 10.1371/journal.pgph.0004764 (PMC12176199; doi:10.1371/journal.pgph.0004764)
Supplement: Attachment — Submitted filename: Point-by-Point Response Letter (Editorss comment).docx.docx [file pgph.0004764.s005.docx]

*31 March 2025*

*Manuscript #: PGPH-D-24-00196*

*TITLE: The effects of Ramadan on clinical consultation frequency in a Rohingya*

*refugee camp, Bangladesh*

**Point-by-Point Response Letter**

Dear Dr. Khan,

Thank you for your thoughtful feedback and for providing us with the opportunity to further refine our manuscript. We sincerely appreciate the time and effort invested by both the editors and reviewers in providing detailed and constructive comments.

In this round of revisions, we have carefully addressed each of the points raised. Specifically, we have provided further justification for our selection of health conditions, clarified our approach to post-natal care visits, and elaborated on our statistical modeling strategy, including the handling of multicollinearity.

Below, we outline our responses to each of the comments, detailing the modifications made to the manuscript accordingly. We hope that these revisions enhance the quality of our work and bring it closer to meeting the publication standards of *PLOS Global Public Health*.

Question

1. **Why did you focus on certain conditions? Is this because the healthcare facility where you collected data provides only these services?**

Our response:

Thank you for raising this important point regarding our focus on ARI, NCDs, gastritis, female-related issues, and injuries. The data collected in the Rohingya camp included a broad range of conditions, such as acute respiratory infections (ARI), skin diseases, gastritis, musculoskeletal disorders, family planning, urinary tract infections, ear problems, oral health issues, conjunctivitis, headaches, helminth infections, injuries, non-communicable diseases (NCDs), general weakness, abscesses, constipation, tonsillitis, diarrhea, fungal infections, general health check-ups, burns, antenatal care (ANC), and postnatal care (PNC). For our analysis, as detailed in our manuscript:

- ARI and NCDs were selected to represent acute and chronic disease patterns.
- Injuries highlighted urgent care needs within the camp.
- Existing literature suggests a link between Ramadan, diet, and gastrointestinal symptoms, making gastritis a relevant condition for analysis.
- Female-related issues were included due to concerns that traditional Rohingya cultural norms might discourage women from seeking ANC and PNC checkups, despite strong recommendations from WHO and the health cluster to promote maternal and neonatal health.

In contrast, conditions such as family planning and oral health were not prioritized, as they do not pose the same level of immediate clinical urgency as ARI, NCDs, and injuries. Other conditions, including general weakness, headache, and musculoskeletal problems, were often nonspecific complaints without clear diagnoses, and thus were excluded from the analysis.

To clarify our rationale, we have modified the method section. We believe that this explanation provides a clear justification for our analytical approach.

2. **The findings you generated for these particular conditions are very much understandable. It is very common for all Muslim people, including Muslims in a normal context, to receive lower healthcare services because they prioritize prayers and related issues. This time-related issue also often comes with religious concerns. For instance, checking for diabetes often involves getting a blood sample, which is not allowed during fasting according to religious beliefs. You recorded a higher number of gastritis patients because fasting can aggravate gastritis symptoms, and seeking treatment for it does not require anything that contradicts religious practices, such as taking a blood sample.**

Our response:

Thank you for your insightful comment. We greatly appreciate your observation regarding the influence of religious practices on healthcare-seeking behavior during Ramadan. We understand that many Muslims may avoid seeking care during fasting hours due to prayer commitments or restrictions such as the reluctance to provide blood samples, particularly in cases like diabetes. This is indeed an important aspect of Ramadan-related health behavior.

We would also like to clarify that our PHC operates with minimal laboratory capacity, and most diagnoses are made clinically without requiring blood sampling. As such, while religious concerns may influence healthcare-seeking behavior in general, we believe that their impact is limited in our setting, where routine blood tests are typically not part of patient care. We have added this explanation to the manuscript to better contextualize our findings.

Additionally, we noted your comment regarding the increased number of gastritis cases during Ramadan. We assume this was the intended condition referenced (rather than arthritis), as our dataset did not include arthritis as a separately analyzed category. Indeed, as mentioned in our Discussion section, the increase in gastritis may be attributed to fasting-related dietary habits and is consistent with previous findings that link Ramadan with digestive issues. We thank you for highlighting this important connection.

3. **I am a bit surprised to see the effects of fasting on post-natal care service access. What could be the reason for such higher odds, given that Rohingya women usually avoid post-natal care unless complications arise? Did you consider post-natal care only for those who delivered at the healthcare facilities where you collected data and received care before leaving the facilities?**

Our response:

Thank you for your insightful question regarding the higher odds of post-natal care (PNC) service access during Ramadan.

At the time of data collection, our facility was the only Primary Health Center (PHC) with an obstetrician in the assigned camp area. Given this context, the health cluster strongly prioritized the promotion of antenatal care (ANC) and PNC, and our clinic actively reinforced these efforts. In particular, our PHC employed two local Rohingya traditional birth attendants who played a crucial role in educating women about the importance of PNC. Additionally, as was common practice in many clinics within the refugee camps at the time, incentives such as food distributions were provided to encourage attendance, which likely contributed to maintaining relatively high PNC visit rates.

It is also important to note that PNC services at our facility were primarily provided to women who had delivered at the clinic. Therefore, the observed trend in PNC visits likely reflects the effectiveness of these targeted initiatives, rather than a substantial change in health-seeking behavior during Ramadan.

We have revised the manuscript to reflect these clarifications.

4. **How many models did you run for Table 4? Was it just one? If so, how did you address multicollinearity? What factors were adjusted?**

Our response:

In our analysis, we conducted separate multivariable logistic regression models, each using a binary independent variable representing the period of PHC visits (BR period vs. Ramadan period, and Ramadan period vs. AR period). The dependent variables analyzed included the type of visit (new or follow-up), procedures performed, patient outcomes, and specific health events such as ARI, NCDs, diarrhea, injuries, antenatal care (ANC), and postnatal care (PNC).

All regression models were adjusted for age and sex to account for potential confounding. Additionally, we tested for interaction effects between age and the Ramadan period as well as between sex and the Ramadan period, but found no significant interactions. As a result, we proceeded with models adjusted solely for age and sex, without including interaction terms.

As our models included only binary or categorical independent variables (e.g., period of PHC visits, age groups, sex), the Variance Inflation Factor (VIF) was not a meaningful measure of multicollinearity. Therefore, we examined the correlation matrix, and found no substantial correlations between the independent variables included in the models.

We have incorporated this clarification into the Methods section.
